# The impact of reconstruction and scanner characterisation on the diagnostic capability of a normal database for [^123^I]FP-CIT SPECT imaging

**DOI:** 10.1186/s13550-016-0253-0

**Published:** 2017-01-24

**Authors:** John C. Dickson, Livia Tossici-Bolt, Terez Sera, Jan Booij, Morten Ziebell, Silvia Morbelli, Susanne Assenbaum-Nan, Thierry Vander Borght, Marco Pagani, Ozlem L. Kapucu, Swen Hesse, Koen Van Laere, Jacques Darcourt, Andrea Varrone, Klaus Tatsch

**Affiliations:** 1 Institute of Nuclear Medicine, University College London Hospital NHS Foundation Trust, 235 Euston Road, London, NW1 2BU UK; 2 Department of Medical Physics, University Hospital Southampton NHS Foundation Trust, Southampton, UK; 3 Department of Nuclear Medicine and Euromedic Szeged, University of Szeged, Szeged, Hungary; 4 Department of Nuclear Medicine, Academic Medical Center, University of Amsterdam, Amsterdam, The Netherlands; 5 Neurobiology Research Unit, Rigshospitalet and University of Copenhagen, Copenhagen, Denmark; 6 Nuclear Medicine Unit, Department of Health Sciences, IRCCS San Martino—IST, University of Genoa, Genoa, Italy; 7 Department of Nuclear Medicine, Medical University of Vienna, Vienna, Austria; 8 Nuclear Medicine Division, Université Catholique de Louvain, Mont-Godinne Medical Center, Louvain-la-Neuve, Belgium; 9 Institute of Cognitive Sciences and Technologies, CNR, Rome, Italy; 10 Department of Nuclear Medicine, Karolinska Hospital, Stockholm, Sweden; 11 Department of Nuclear Medicine, Faculty of Medicine, Gazi University, Ankara, Turkey; 12 Department of Nuclear Medicine, University of Leipzig, Leipzig, Germany; 13 Division of Nuclear Medicine, University Hospital and K.U. Leuven, Leuven, Belgium; 14 Nuclear Medicine, Centre Antoine Lacassagne and University Hospital, Université de Nice de Sophia Antipolis, Nice, France; 15 Department of Clinical Neuroscience Psychiatry Section, Karolinska Institute, Stockholm, Sweden; 16 Department of Nuclear Medicine, Municipal Hospital of Karlsruhe Inc., Karlsruhe, Germany

**Keywords:** [^123^I]FP-CIT, SPECT, Quantification, Reconstruction, Dopamine transporter

## Abstract

**Background:**

The use of a normal database for [^123^I]FP-CIT SPECT imaging has been found to be helpful for cases which are difficult to interpret by visual assessment alone, and to improve reproducibility in scan interpretation. The aim of this study was to assess whether the use of different tomographic reconstructions affects the performance of a normal [^123^I]FP-CIT SPECT database and also whether systems benefit from a system characterisation before a database is used.

Seventy-seven [^123^I]FP-CIT SPECT studies from two sites and with 3-year clinical follow-up were assessed quantitatively for scan normality using the ENC-DAT normal database obtained in well-documented healthy subjects. Patient and normal data were reconstructed with iterative reconstruction with correction for attenuation, scatter and septal penetration (ACSC), the same reconstruction without corrections (IRNC), and filtered back-projection (FBP) with data quantified using small volume-of-interest (VOI) (BRASS) and large VOI (Southampton) analysis methods. Test performance was assessed with and without system characterisation, using receiver operating characteristics (ROC) analysis for age-independent data and using sensitivity/specificity analysis with age-matched normal values. The clinical diagnosis at follow-up was used as the standard of truth.

**Results:**

There were no significant differences in the age-independent quantitative assessment of scan normality across reconstructions, system characterisation and quantitative methods (ROC AUC 0.866–0.924). With BRASS quantification, there were no significant differences between the values of sensitivity (67.4–83.7%) or specificity (79.4–91.2%) across all reconstruction and calibration strategies. However, the Southampton method showed significant differences in sensitivity between ACSC (90.7%) vs IRNC (76.7%) and FBP (67.4%) reconstructions with calibration. Sensitivity using ACSC reconstruction with this method was also significantly better with calibration than without calibration (65.1%). Specificity using the Southampton method was unchanged across reconstruction and calibration choices (82.4–88.2%).

**Conclusions:**

The ability of a normal [^123^I]FP-CIT SPECT database to assess clinical scan normality is equivalent across all reconstruction, system characterisation, and quantification strategies using BRASS quantification. However, when using the Southampton quantification method, performance is sensitive to the reconstruction and calibration strategy used.

## Background

Imaging of the dopaminergic transporter in the striatum using [^123^I]FP-CIT SPECT (or ^123^I-ioflupane, marketed as DaTSCAN) is a well-established technique to assist in the diagnosis of Parkinson’s Disease (PD) [1] and Lewy body dementia (DLB) [2]. In a substantial number of studies, visual assessment is adequate to accurately interpret images; however, with difficult to interpret cases, quantification of striatal tracer binding can be beneficial in coming to a more confident interpretation of the scan [3, 4] or to increase confidence in less-experienced readers [5].

In recent years, the Neuroimaging Committee of the European Association of Nuclear Medicine put together a multi-centre group to create a substantial database of normal healthy control subjects with [^123^I]FP-CIT imaging over a wide age range [6]. The result of the project was the ENC-DAT database of 140 subjects created using 16 scanners, which gave both visual and quantitative information on striatal dopamine transporter availability in healthy subjects between 20 and 83 years of age. To achieve the most accurate quantitative values possible, tomographic data was iteratively reconstructed with corrections for photon attenuation, photon scatter and septal penetration. Although this worked well to help achieve the aims of the project, for wider dissemination of the database throughout the nuclear medicine community, the use of corrections has proved to be problematic. Attenuation correction of brain SPECT data is frequently performed in the nuclear medicine community using the Chang attenuation correction method [7]. However, this method is challenging with dopamine transporter imaging because outlining the brain (a part of the attenuation correction process) is difficult (low binding due to low DAT expression in cortical areas) and often inconsistent without specialist software [8]. Correction for scatter and septal penetration is also problematic, because the frequently used triple energy window method [9] applied for the database requires energy window definition and image processing steps that may not be accessible to all sites. Furthermore, in addition to these corrections, the iterative reconstruction algorithm itself is also not available or indeed preferred at some centres.

An additional challenge for disseminating the database has been the requirement to perform a detailed phantom calibration to define normal ranges for a specific imaging system [10]. This strategy was implemented because the imaging characteristics of gamma cameras are different, even within the same scanner type. Small changes in spatial resolution, sensitivity, and the degree of collimator septal penetration to higher energy Iodine-123 emissions all have an effect on the overall quality and quantitative ability of a gamma camera system. By performing an anthropomorphic phantom calibration of known striatal and background activity concentrations, it is possible to characterise the performance of individual imaging systems and derive normal ranges specific to the gamma camera in question. Unfortunately, this camera characterisation is not trivial and requires an expensive anthropomorphic phantom, which has limited its use in individual centres.

With such challenges, this paper will explore whether the ENC-DAT database is as powerful at aiding [^123^I]FP-CIT scan interpretation using iterative reconstruction performed without corrections for attenuation and scatter/septal penetration. The performance of the database using a more widespread filtered back projection reconstruction (again without corrections) will also be assessed. Additionally, this work will determine whether a phantom calibration to produce a system specific normal range is required. Such assessments will be made by comparing the diagnostic performance of quantification with different reconstructions, with and without derived camera specific normal ranges.

## Methods

### Subject data

To create the normal database, 123 subjects from the ENC-DAT project were used in this work [6]. Data was omitted if it was not acquired on a gamma camera with parallel hole collimators, had no scatter energy windows or had other technical issues. This involved data from 11 different sites and gamma cameras. To assess the diagnostic ability of the normal databases, 77 subjects from two sites (London and Southampton) who had 3-year follow-up information were used, with their working clinical diagnosis acting as our standard of truth. Of the 77 subjects, 16 subjects were referred for an AD/DLB differential diagnosis, 14 were referred for atypical parkinsonian syndromes while the rest (47) were referred for helping to establish whether the patient had Parkinson’s disease. All patients were scanned according to the protocol defined in [6].

### Data processing and analysis

Subject data were reconstructed using three different reconstruction algorithms: the iterative reconstruction with filtering and corrections for attenuation and septal and scatter penetration (ACSC) recommended by the ENC-DAT project [10]; the same iterative reconstruction without corrections (IRNC); and uncorrected filtered back projection (FBP). All data was filtered using a Butterworth filter (cutoff 0.55 cm^−1^, power factor 10). Following reconstruction, image data was quantified with Hermes BRASS [11] and Southampton [12] methods.

Using a phantom calibration technique [10], the quantified ENC-DAT data was pooled in terms of “true” striatal/caudate/putaminal specific binding ratios (SBR). In short, a set of phantom data from each scanner involved in the ENC-DAT project was reconstructed using each reconstruction method, so that linear relationships of measured vs true phantom SBRs could be derived. Using these linear relationships, normal subject data from each scanner could be reconstructed using the different techniques and pooled in terms of true uptake to produce three sets of quantitative data (ACSC; IRNC; FBP). With the data in this form, a negative linear relationship was assumed between striatal SBR and age so that a fit could be applied together with upper and lower bounds defined as twice the standard error of the predicted *y* value for each *x* in the regression. The lower of these lines acted as the threshold of normality.

Using the patient’s clinical records, the working clinical diagnosis of 77 subjects with 3-year follow-up acted as the standard of truth for this study (Table 1). With the phantom-based calibration just described, the quantitative SBR from the follow-up studies were converted to true striatal SBR before being compared to the lower limits of the normal database. This was done for each of the three reconstruction techniques. Using the BRASS technique, abnormality was determined using two mechanisms: based on whether the SBR in a single striata was abnormal and based on whether the SBR in a single putamen was abnormal. This is because for a given patient, if at least one side of the brain is classified as abnormal, then the scan is deemed to be abnormal. Using the Southampton method, scan abnormality was based on striatal SBRs only.

**Table 1:** Demographics and expected [^123^I]FP-CIT SPECT findings of 3-year follow-up subjects

Centre	Mean age (range)	Total	Female/male	Abnormal/normal
London	62.8 (25–84)	44	21/23	21/23
Southampton	65.9 (49–84)	33	16/17	22/11
All	63.4 (25–84)	77	37/40 (48%/52%)	43/34 (55%/45%)

To assess whether a phantom scan is required for scanner characterisation, a similar process as described above was performed on non-calibrated database and follow-up data. That is, the ‘raw’ SBR values from the quantification software were used ‘as is’ and were not converted into true SBR using the phantom measurements.

### Statistics

Diagnostic performance was assessed at two levels.

1. To determine the overall diagnostic performance of software using different reconstruction and calibration strategies, receiver operating characteristics (ROC) analysis was performed to derive the areas under the curve (AUC) and their associated 95% confidence intervals. Significance of differences between strategies was assessed using the method proposed by [13].
2. Using the derived normal limits, age-stratified performance of the database was determined using sensitivity and specificity analysis with comparisons in sensitivities and specificities performed using a two-tailed McNemar chi-square test.

In all statistical comparisons, 3-year clinical follow-up acted as the standard of truth. Analysis was performed using GraphPad Prism v6.0 (http://www.graphpad.com/), with statistical significance set at *p* < 0.05.

## Results

### Overall quantitative performance

The results of ROC analysis assessing the overall diagnostic performance of the different reconstruction and calibration strategies are shown in Table 2. There is no significant difference in overall diagnostic performance between the different reconstruction strategies, although the highest AUC is seen with putaminal SBR using BRASS software. There is also no difference in the performance of the quantitative values from either software when using or not using a phantom calibration.

**Table 2:** ROC area under the curve (AUC) data together with sensitivity and specificity performance for Southampton-specific striatal binding ratios (SBR) and BRASS striatal and putaminal SBR

		Calibrated			Non-calibrated		
		ACSC	IRNC	FBP	ACSC	IRNC	FBP
ROC (AUC)	BRASS striatum	0.901	**0.916**	*0.884*	0.904	0.915	0.897
	BRASS putamen	0.915	**0.924**	*0.897*	0.919	0.921	0.906
	Southampton	**0.876**	*0.866*	**0.876**	0.874	*0.866*	0.874
Sensitivity	BRASS striatum	83.7	**86.1**	83.7	79.1	*67.4*	74.4
	BRASS putamen	**83.7**	**83.7**	81.4	**83.7**	*76.7*	81.4
	Southampton	**90.7**	76.7	67.4	65.1	67.4	*60.5*
Specificity	BRASS striatum	85.3	82.4	*79.4*	85.3	**91.2**	88.2
	BRASS putamen	82.4	85.3	*79.4*	85.3	**91.2**	88.2
	Southampton	*82.4*	*82.4*	*82.4*	**88.2**	**88.2**	**88.2**

The table shows the results for data with and without using a phantom calibration for iterative reconstruction with attenuation and scatter/septal penetration reconstruction (ACSC), iterative reconstruction with no corrections (IRNC) and filtered back projection reconstruction, again with no corrections (FBP). The best performance measure for each quantification technique is in bold, while the weakest is shown in italics

### Performance based on age-stratified normal ranges

**Fig. 1:**
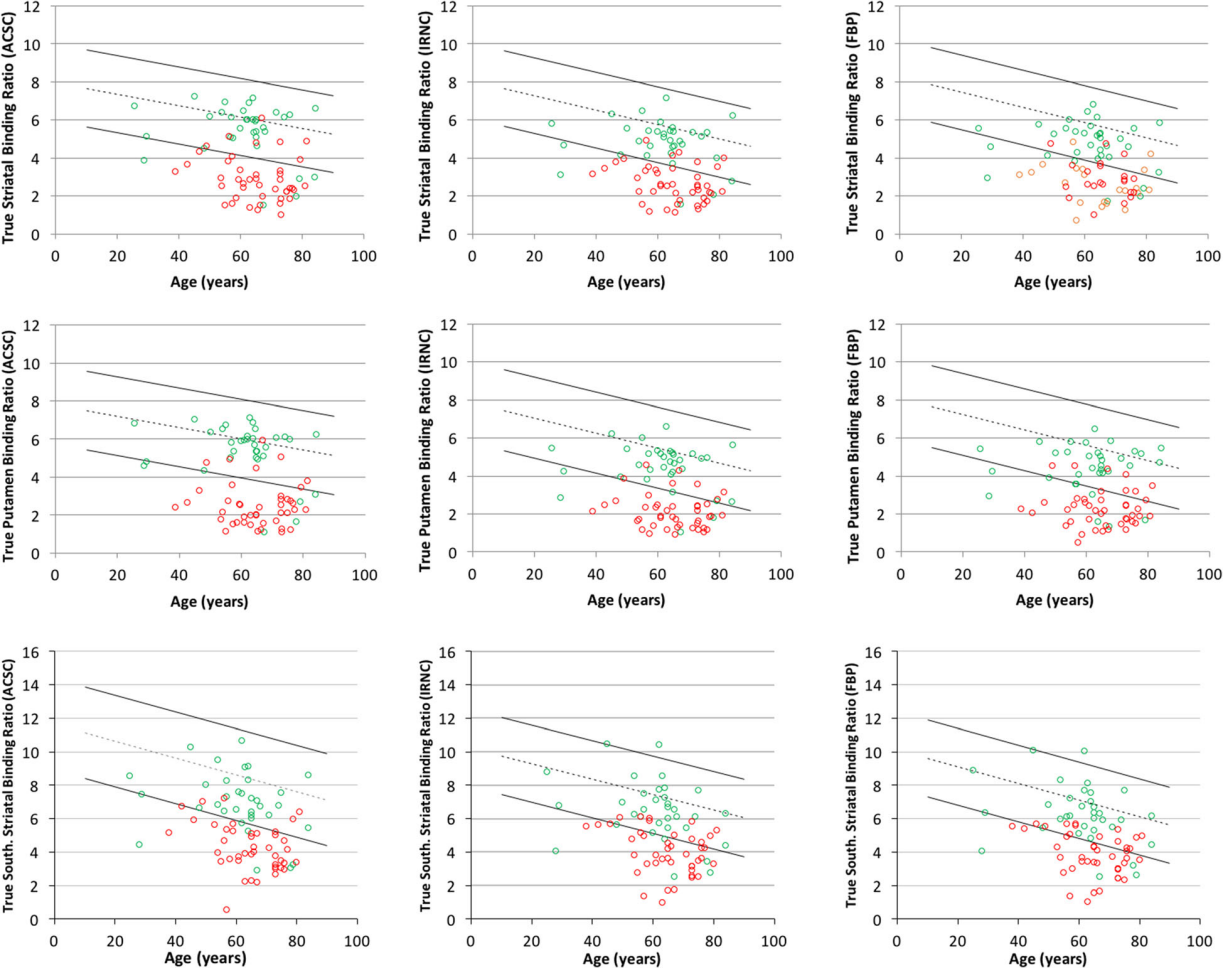
Calibrated normal ranges with follow-up data. Age-dependent ‘normal’ ranges of different calibrated quantitative SBRs measures with various reconstructions. The *dashed line* shows the linear fit of the normal data, with *solid lines* representing the upper and lower level normal range given by 95% confidence levels of the mean. *Coloured dots* represent patients suffering (*red*) and not suffering (*green*) from a syndrome characterised by dopaminergic degeneration based on 3-year follow-up

**Fig. 2:**
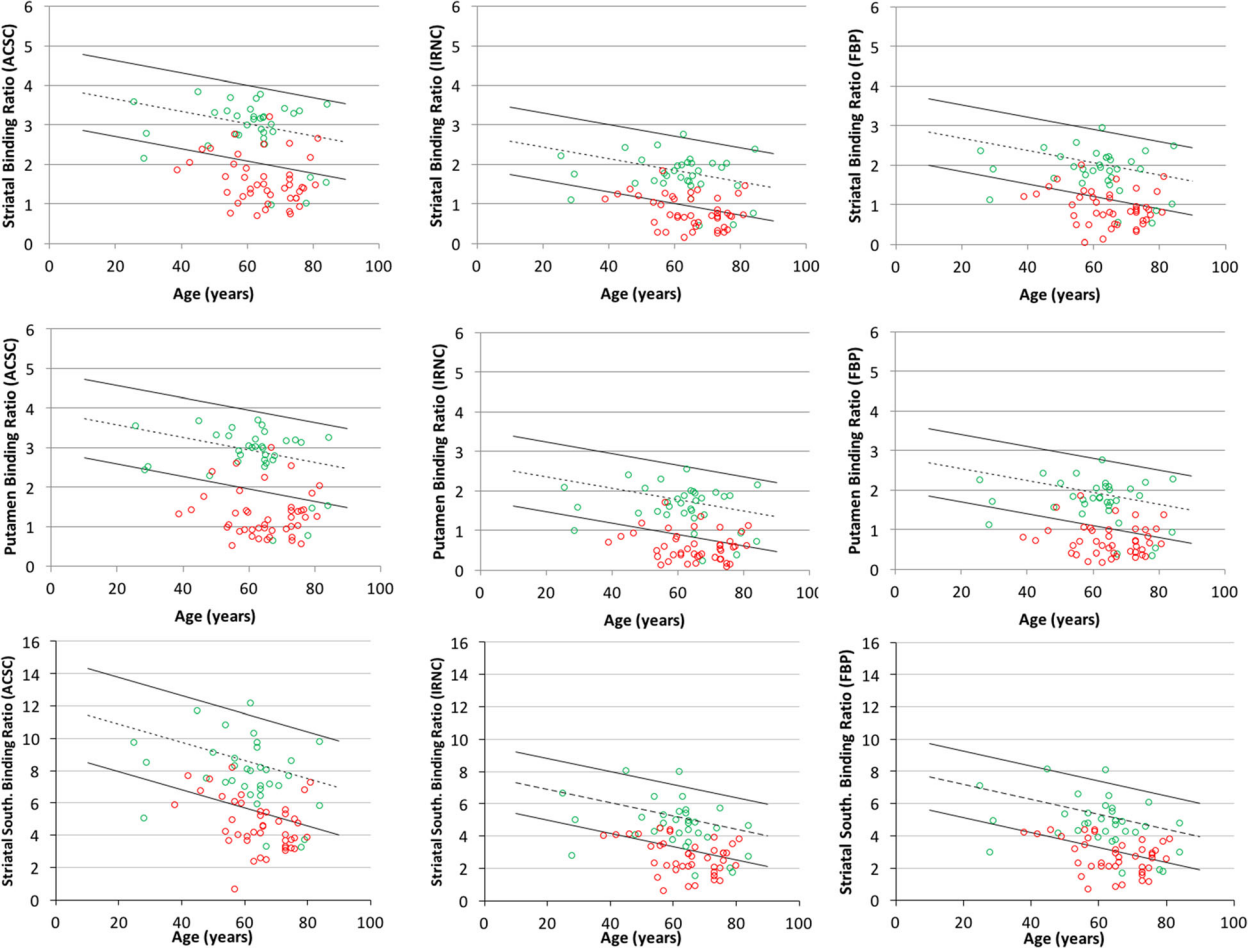
Non-calibrated normal ranges with follow-up data. Age-dependent normal ranges of different non-calibrated quantitative measures with various reconstructions. The *dashed line* shows the linear fit of the normal data, with *solid lines* representing the upper and lower level normal range given by 95% confidence levels of the mean. *Coloured dots* represent patients suffering (*red*) and not suffering (*green*) from a syndrome characterised by dopaminergic degeneration based on 3-year follow-up. Note the different *y*-axis ranges

The results from sensitivity and specificity analysis using age-stratified normal ranges are also shown in Table 2. Example plots showing the distribution of normal data with ranges, and the follow-up patients are given in Figs. 1 and 2 with the number of incorrectly classified false positive and false negative data shown in Table 3.

**Table 3:** The number of false positive and false negatives recorded for the different quantification methods, reconstruction techniques and calibration strategies

		Calibrated			Non-calibrated		
		ACSC	IRNC	FBP	ACSC	IRNC	FBP
False positives (34 normal subjects)	BRASS striatum	5	6	7	5	3	4
	BRASS putamen	6	5	7	5	3	4
	Southampton	6	6	6	4	4	4
False negatives (43 abnormal subjects)	BRASS striatum	7	6	7	9	14	11
	BRASS putamen	7	7	8	7	10	8
	Southampton	4	10	14	14	14	17

Using BRASS with either striatal or putaminal SBRs, there was no significant difference between the values of sensitivity and specificity across all reconstruction and calibration strategies although there were subtle differences in false negative rates using non-calibrated data, particularly for striatal SBR. Conversely, with the Southampton quantification method, although specificity remained similar across reconstructions and calibration strategies (82.4–88.2%), there were areas with significant changes in sensitivity. Comparisons across calibrated and non-calibrated data showed significant (*p* = 0.002) improvements in the performance of ACSC data with calibration, with sensitivity increasing from 65.1 to 90.7% and false negative numbers changing from 14 to 4. Other reconstructions showed slightly smaller, non-significant changes (IRNC 67.4 to 76.7%, FBP 60.5 to 67.4%), with false negative numbers changing from 14 to 10 (IRNC) and 17 to 14 (FBP). Assessments across reconstruction methods showed significantly better sensitivity in calibrated ACSC reconstructed data (90.7%) than both IRNC at 76.7% and FBP at 67.4% (*p* = 0.03 and 0.002, respectively). False negative numbers changed from 4 when using ACSC to 10 for IRNC and 14 for FBP. No differences were found in non-calibrated data.

Comparing analysis methods, calibrated FBP data had significantly lower sensitivity with the Southampton method (67.4%) with 14 false negatives compared to striatal BRASS SBRs at 83.7% and 7 false negatives (*p* = 0.04). With non-calibrated data, putamen BRASS SBR was significantly more sensitive than the Southampton method for both ACSC (83.7 and 65.1%) and FBP reconstructions (81.4 and 60.5%) with *p* = 0.04 and 0.01, respectively (false negative figures are shown in Table 3).

**Fig. 3:**
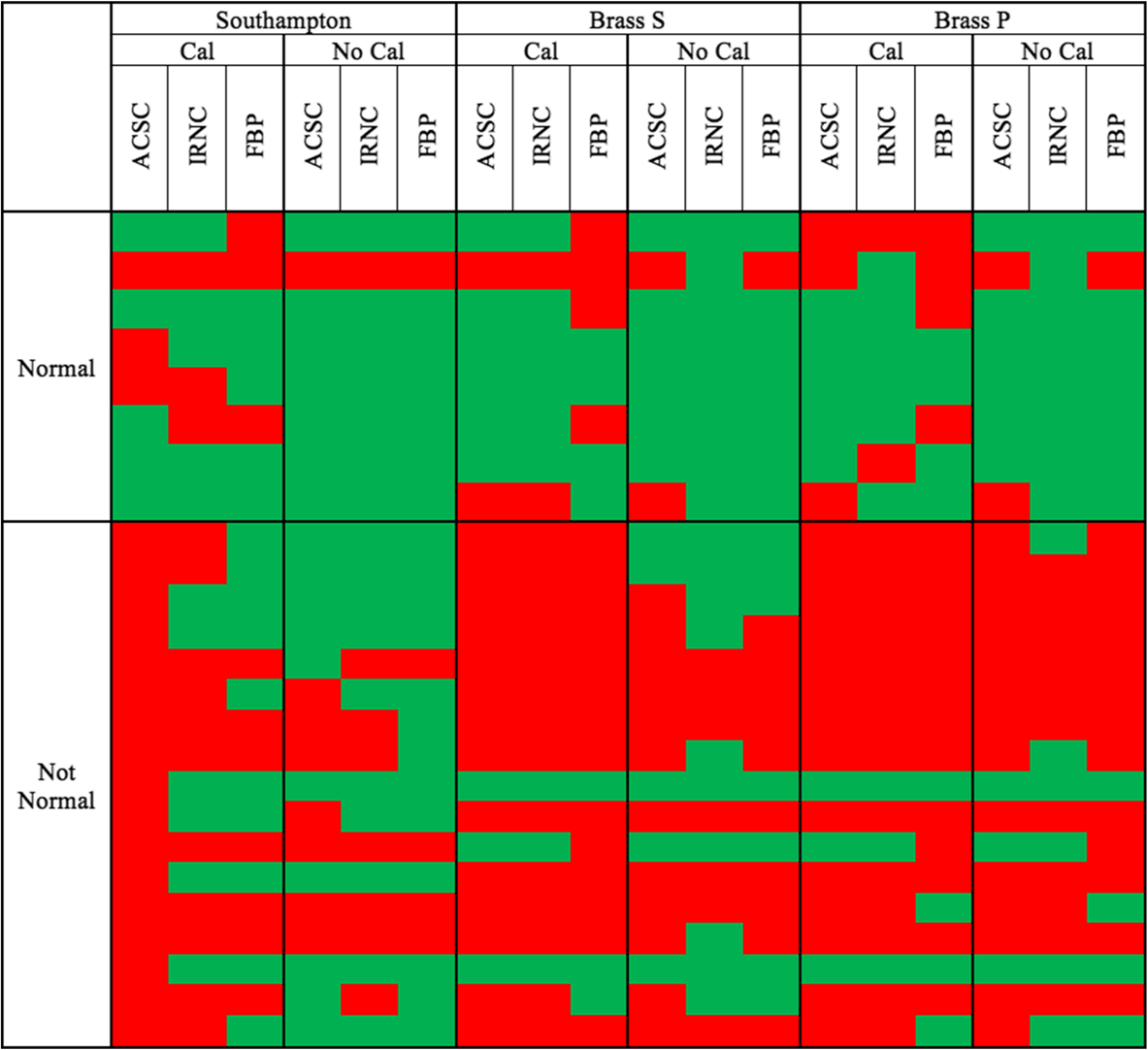
Discordance quantitative diagnoses. Cases with discordant quantitative diagnoses. Each row is grouped into cases with a clinically normal and abnormal diagnosis. For a specific quantification, reconstruction and calibration strategy, quantitatively normal diagnoses are shown in *green*, while an abnormal quantitative diagnosis in shown in *red*

In Figs. 1 and 2, it is clear that some borderline cases jump from normality to abnormality and vice-versa with different reconstructions and calibration strategies, which explains the differing sensitivity and specificity results. The fact that the magnitude and age relationship of normal values changes with the approach taken adds to why the quantitative diagnosis changes in some studies. These changing quantitative diagnoses, and the resulting reconstruction- and calibration-related discordance between clinical and quantitative assessments, is summarised in Fig. 3. For clinically normal subjects, there was no clear underlying trend except for quantification derived from calibrated FBP data being more likely to disagree with clinical diagnosis. For clinically abnormal cases, two patients showed an abnormal quantitative diagnosis with the calibrated ACSC data using the Southampton SBR method that were not quantitatively abnormal with any other method. The Southampton method, and also to a lesser extent the use of BRASS striatal volume-of-interests (VOIs), displayed an improved performance with system calibration.

## Discussion

Quantification of striatal [^123^I]FP-CIT SPECT binding can prove useful for clinical reporting, to assist in difficult to interpret cases and to help less-experienced reporters with their image interpretation. However, the normative values from healthy subjects required to contextualise the results are dependent on the reconstruction algorithm and scanner used. In this study, we have explored how different reconstructions affect the diagnostic ability of quantitative values of striatal SBRs and also investigated whether scanner characterisation is required to make the best use of a multiple scanner-derived normal database.

When assessing the diagnostic performance of all SBRs without age stratification using ROC analysis (Table 2), there were no significant differences across each of the investigated reconstructions. Furthermore, without age stratification, scanner calibration to derive scanner-specific normal ranges was also shown to have no significant benefit. On closer investigation, there is a tendency for the performance of putaminal SBRs with BRASS to perform slightly better than the assessment of full striatal SBRs with the same software, which in turn performs slightly better than the Southampton method of quantification. Of course, this finding depends in part on our follow-up cohort; however, it is of no surprise with this group of patients that putaminal SBRs would perform better than an assessment of striatal SBRs given that idiopathic Parkinson’s disease has a tendency to affect the putamen first. When relying on striatal SBR measurements alone, there are instances when reduced putaminal SBR may be dominated by a high SBR in the caudate nucleus resulting in a normal overall measurement of striatal SBR.

Since there is a known age-related decline in tracer uptake [6], age-stratified diagnostic performance was assessed using sensitivity/specificity analysis (Table 2). Using a calibration to derive system specific normal ranges provided no significant benefit for BRASS striatal or putamen SBRs. There was a trend for slightly lower striatal BRASS sensitivities and higher numbers of false negatives without calibration, but these were not significant. Conversely, with the Southampton method of deriving SBRs, calibration led to increased sensitivity, a trend present in the FBP and IRNC reconstructions that became significant for the ACSC data. The impact of this is seen in the reduced false negative numbers with calibration shown in Table 3. Specificity remained similar with or without calibration across all reconstructions and software. Knowing that the true negative rate is similar across strategies is helpful given that specificity is very important in the interpretation of this test and arguably more important than sensitivity given this test is not used as a screening tool in clinical use.

**Fig. 4:**
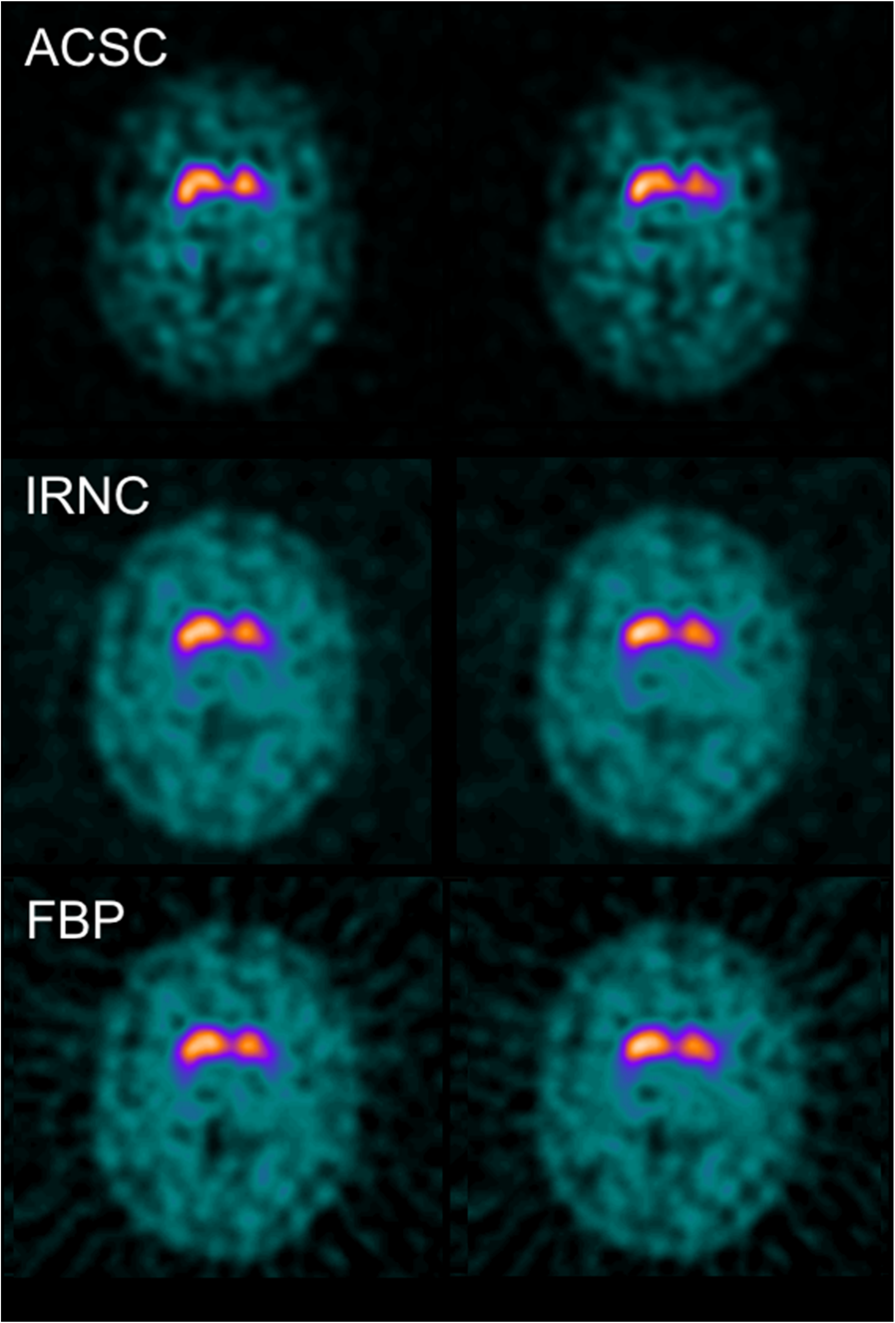
Divergent quantitative diagnosis based on calibration. Two consecutive slices from a study of a 46-year-old female patient with a 3-year follow-up diagnosis of a dopaminergic degenerative disorder (Parkinson’s disease), which has a divergent quantitative diagnosis based on the use of calibration. Without calibration, the study was frequently found to be normal, while with calibration the study was mostly abnormal

To understand the differences caused by calibration, we must try to understand what this system characterisation is achieving. The primary loss in signal from this type of imaging is through partial volume effects. Using the BRASS method of quantification where tight VOIs are particularly susceptible to partial volume effects, the primary influence of the calibration is to correct for these effects, as can be seen in the pre- and post-calibration normal ranges with this method (Figs. 1 and 2, respectively). Changes in striatal size are modest across patients and indeed the normal subjects [14], at least when compared to system spatial resolution [15]. Given that the spatial resolution of most modern SPECT systems is relatively similar with clinical radius of rotations and typical reconstruction filters, the partial volume effects for individual subjects will remain relatively consistent. As a consequence, the calibration has little effect on diagnostic performance of BRASS quantification. A small reduction in performance is found with non-calibrated striatal SBR measures which is likely caused by the partial volume effects of the smaller caudate nuclei when it is included in the VOI. This is indeed what happens on the left side of the brain in the study shown in Fig. 4. Conversely with the Southampton method of quantification, partial volume factors are already accounted for, which can be seen by the much smaller differences in calibrated and non-calibrated values (Figs. 1 and 2). The calibration for the Southampton method is therefore primarily correcting for other scanner effects such as septal penetration from intra-brain and extra-brain activity (i.e. from salivary glands and distant organs), which can be more variable across scanner types (Tossici-Bolt in submission). As a result, calibration for this form of quantification becomes more important.

The use of different reconstructions has little effect on the diagnostic performance of the BRASS quantification. We might expect performance to be equalised with calibration; however, the delivery of similar performance across reconstructions using non-calibrated data does at first seems surprising. We would expect that the attenuation of I-123 gamma photons would be relatively consistent across subjects and also that the scatter environment would be similar too. Septal penetration, which we hope to be reduced using scatter correction, does differ across subjects scanned on different scanners (Tossici-Bolt in submission) but the results here suggest that this is not a major factor with BRASS quantification. Conversely, with the Southampton method, there are again significant differences in sensitivity between ACSC and IRNC reconstructions and ACSC and FBP reconstructions using calibrated data. Specificity remains unchanged. Once more, with the partial volume factors removed using this quantification method, the corrections for attenuation and scatter become more relevant. With non-calibrated data, any gain in diagnostic performance using corrections is lost, and differences between reconstructions no longer seen.

**Fig. 5:**
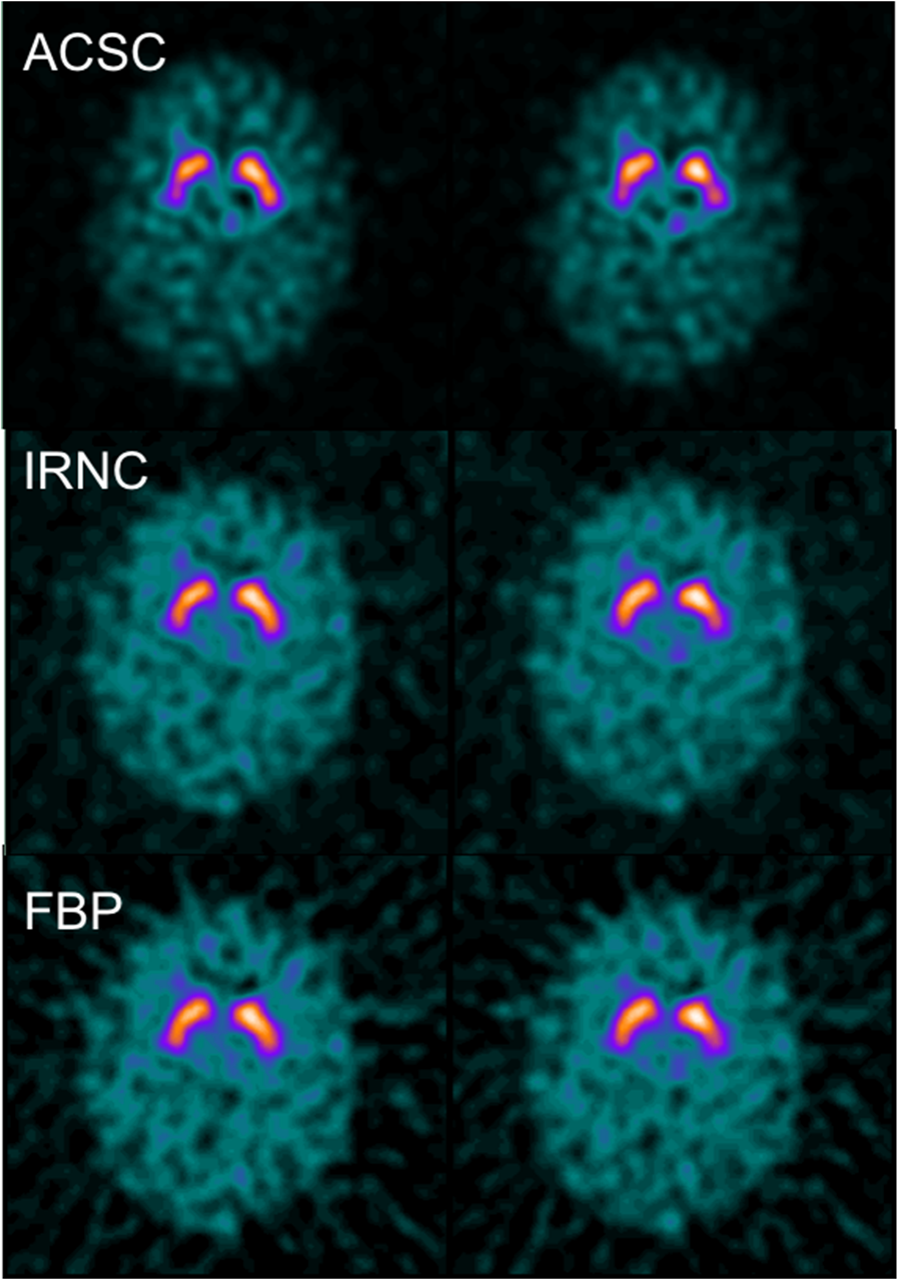
Divergent quantitative diagnosis based on reconstruction. Two consecutive slices from a study of a 29-year-old female patient deemed not to have a degenerative dopaminergic syndrome on follow-up, with divergent quantitative diagnosis depending on the reconstruction used. Using a FBP reconstruction with calibration, quantification found the study to be abnormal. Using other reconstructions, the scan was rated as normal

We can see changes in quantitative diagnosis by focussing on borderline cases. Figure 5 shows a clinically normal follow-up subject whose diagnosis changes based on the reconstruction used. When reconstructed using FBP with calibration, this study is found to be abnormal, while for most other reconstructions with or without calibration, the study is quantitatively normal. Visually, this study looks normal, although a mild reduction in DAT binding is seen in the left posterior putamen and possibly on the right posterior putamen as seen in the ACSC reconstruction. Figure 3 shows that clinically normal cases disagree quantitatively most often with calibrated filtered back projection reconstruction. Compared to FBP, the iterative reconstruction suffers two disadvantages: its spatially varying convergence rates and its non-negativity constraint [16]. The non-negativity constraint is likely to elevate count density in the presence of low count densities, while the convergence rates of iterative reconstruction are likely to lead to underestimations of count densities since incomplete convergence is the norm in nuclear medicine imaging. Both these factors would reduce SBRs potentially making a normal study abnormal. However, in this instance, the opposite effect is seen with FBP reconstruction leading to an abnormal outcome while other reconstructions give a normal outcome. In Fig. 1, it can be seen that FBP produces slightly and indeed significantly (Tossici-Bolt in submission) higher values of striatal SBRs in the normal range compared to IRNC, which means for this individual borderline case, the increase in value is not as great as the average for the normal range. As a consequence, this small shift in quantitative value has led to a large shift in diagnosis from normal to abnormal, reminding us that the quantitative assessment of borderline cases either below or above the ‘normality line’ should be treated with caution if these values are to guide clinical interpretation.

**Fig. 6:**
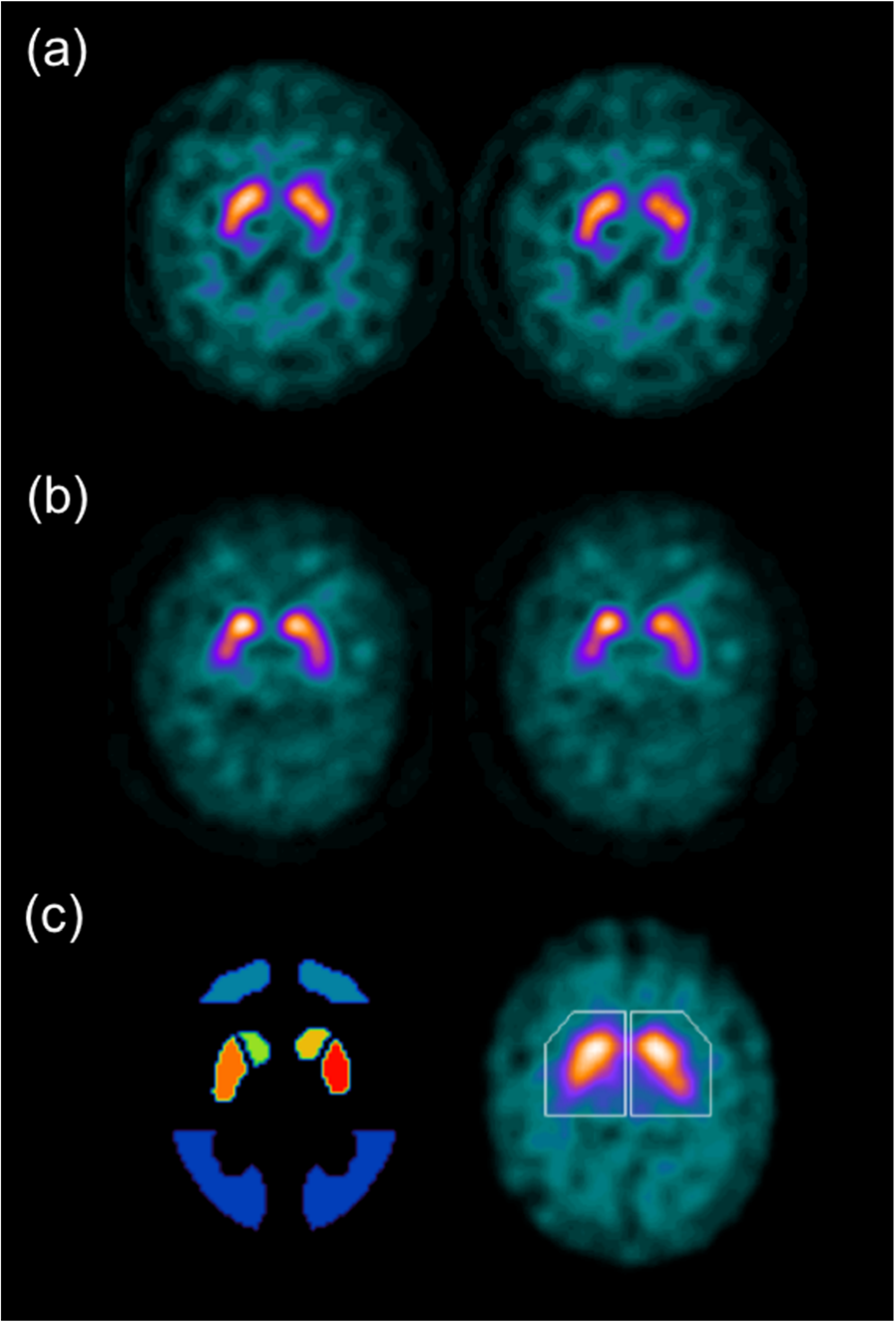
Divergent quantitative diagnosis based on method of quantification. Patient studies with divergent quantitative diagnosis based on the quantification method used. **a** Two consecutive slices from a study of a 73-year-old male patient with a working diagnosis of a dopaminergic degenerative disorder (Lewy body dementia) are found to be abnormal using all BRASS quantification and rated normal in all but the calibrated ACSC reconstruction using Southampton quantification. **b** A clinically abnormal study of a 65-year-old female patient with a 3-year follow-up diagnosis of a dopaminergic degenerative disorder (multiple system atrophy) which was normal using BRASS quantification with the exception of striatal and putaminal binding ratios using a calibrated FBP approach (and putamen non-calibrated) and rated abnormal with the Southampton method of quantification. **c** The different assessment regions used. On the *left* is one slice of several highlighting tight BRASS striatal regions of interest over the caudate and putamen in addition to the occipital lobe reference region. *Right*, the Southampton method is applied to summed striatal slices with large VOIs to account for partial volume effects with the reference region encompassing all other brain pixels

Comparing the diagnostic performance of the quantification methods, the Southampton method with ACSC reconstructed data and calibration has a slightly higher sensitivity than all other methods. This is highlighted in Fig. 3, where two clinically abnormal cases that were only classified as quantitatively abnormal using this methodology, although statistically this method wasn’t significantly better than many of the alternative techniques. There are however some significant differences in performance. Calibrated striatal BRASS quantification with FBP reconstruction has a higher sensitivity than the Southampton method because of the reduced performance of the FBP method with this form of quantification as described above. In terms of non-calibrated data, the poor performance of the Southampton method under these conditions is reflected in poorer sensitivity than putamen BRASS measures with both ACSC and FBP reconstructions. An example of discordance from quantification method is given in the clinically abnormal follow-up studies shown in Fig. 6. In Fig. 6a, this study with visually balanced loss, clinically rated as abnormal, is found to be abnormal quantitatively using all BRASS measures and normal using almost all Southampton measures. While the Southampton method’s large VOI approach is helpful to help account for partial volume losses, it can create problems in the small number of patients with dilated ventricles, as shown in this Figure. If the proportion of ventricle in the striatal and background areas is balanced, then quantification is not affected. However, if there is an imbalance of ventricular volumes in these regions, the SBR can be over- or under-expressed. Conversely, in some instances, the larger background region of interest used in the Southampton method can add benefit by reducing noise and therefore uncertainty in the measurement of non-specific uptake [17]. In Fig. 6b, we see an incorrect quantitative diagnosis using BRASS methods, while the Southampton method concords with the follow-up clinical diagnosis. Uptake in the caudate nuclei looks good in this study, which explains the normal interpretation based on striatal SBRs, while the anterior putamen still shows good DAT binding and the posterior putamen poorer binding. Overall, the anterior putamen dominates the quantitative uptake figure, even though there is a gradient of reduced uptake as we move to the posteriorly that could be indicative of an abnormal scan. If the BRASS method split the putamen uptake into smaller anterior and posterior uptake figures, the posterior putamen may well have indicated an abnormal scan. In this case, the complete striatal DAT binding figure is below normal limits so that Southampton method gives the interpretation of an abnormal scan, which corresponds to the clinical diagnosis.

This study has focussed on the use of a normal database in a clinical environment; however, there is growing popularity of [^123^I]FP-CIT SPECT imaging in therapeutic research both as a tool to determine entry to a study and also to follow dopamine transporter availability longitudinally through a treatment cycle. For entry to a therapeutic trial, the requirements are similar to what is required for a clinical diagnosis of Parkinson’s disease or Lewy body dementia, although for borderline normal/abnormal cases the implication of an incorrect diagnosis may be different. For longitudinal imaging within a trial or indeed for clinical follow-up, the focus is elsewhere, with more emphasis on repeatability and the sensitivity to detect change. It is not clear whether calibration would be better in this instance. In an ideal scenario, longitudinal imaging would be performed on the same scanner, but this is not always possible. Unfortunately, the influence of calibration, reconstruction and quantification method for longitudinal imaging was not addressed in this project but would be an interesting area of further study.

A potential limitation of this study is that follow-up working diagnosis may be influenced by the result of [^123^I]FP-CIT SPECT imaging. While initially the result of this imaging study will clearly influence working diagnosis, a working diagnosis at 3 years’ follow-up was chosen so that any advance in disease would give a clearer patient diagnosis less influenced by imaging results. Another possible limitation is that for an imaging study that is compromised by the limited spatial resolution of SPECT systems, we have not considered the use of resolution modelling techniques in the image reconstruction comparison. The reason for this is twofold. Firstly, our intention was to move away from complex reconstruction algorithms to those more widely available in the community, and resolution modelling techniques are not standard on most SPECT installations. The second reason for not assessing resolution modelling techniques was because of the known overshoot artefacts arising from this method which can provide difficulties in quantitative imaging [18].

The values of sensitivity and specificity given in this paper are lower than that normally reported for [^123^I]FP-CIT SPECT [3]. This is a likely reflection of the cohort of patients included in this study that were mixed in nature, including typical and atypical parkinsonian syndromes, and patients with Lewy body dementia. An additional factor was that many of the referrals were from a national movement disorder centre and may therefore constitute a more complex mix of patients than that seen in typical imaging centres.

## Conclusions

In conclusion, in this relatively small patient cohort of 77 subjects, this study has shown that although the diagnostic ability BRASS methods of quantification are unaffected by reconstruction and calibration strategy, the Southampton method, which offers greater accuracy by accounting for the dominating issue of partial volume effects, is affected by the choice of both reconstruction and calibration. The choice of quantification method used depends on user requirements. In terms of diagnostic capability, the performance of BRASS quantification is found to be equal to the Southampton method in this cohort and offers the benefit of robustness against reconstruction or calibration choices. If, however, calibration is performed and the Southampton method is used with ACSC reconstruction, not only is diagnostic ability slightly improved but also the accuracy of uptake in SBR measured across the striata becomes a more accurate reflection of the true value.
